# Impact of Desloratadine on Symptoms and Quality of Life in Subjects with Chronic Idiopathic Urticaria: A Multicenter, Practice-based Study

**DOI:** 10.1111/j.1753-5174.2008.00010.x

**Published:** 2008-09

**Authors:** Harold Kim, Charles Lynde

**Affiliations:** * McMaster University Faculty of Health SciencesHamilton, Ontario, Canada; † The University of Western Ontario Schulich School of Medicine & DentistryLondon, Ontario, Canada; ‡ University of Toronto Faculty of Medicine and University Health Network, Toronto Western DivisionToronto, Ontario, Canada

**Keywords:** Chronic Idiopathic Urticaria, Desloratadine, Quality of Life

## Abstract

**Background:**

Controlled trials have demonstrated the efficacy of antihistamines in the treatment of chronic idiopathic urticaria. Second-generation antihistamines are recommended as first-line therapy for chronic idiopathic urticaria. The purpose of this study was to determine the effect of desloratadine, a newer, nonsedating, second-generation antihistamine, on symptoms of chronic idiopathic urticaria, disease severity, and quality of life (QoL).

**Methods:**

In an open-label, observational, multicenter study, 348 subjects with chronic idiopathic urticaria were given 5 mg of desloratadine once daily for 2 weeks. Outcome measures included change from baseline at Day 14 using the Aerius Quality of Life Questionnaire (AEQLQ); change from baseline in pruritus score, number and maximum size of hives, sleep quality, and activity impairment; and subjects' response to therapy.

**Results:**

Desloratadine significantly decreased subjects' overall condition and symptom scores from baseline to Day 14 (2.19 ± [SD] 0.66 and 1.14 ± 0.89, respectively; *P* < 0.0001). Desloratadine treatment significantly improved all 10 AEQLQ domain scores from baseline to Day 7 and Day 14 (*P* < 0.0001). Sleep disturbance scores decreased 40% from baseline to Day 7 (1.42 ± 1.03 to 0.85 ± 0.89, respectively), and interference with daily outdoor activity scores showed a 41% decrease from baseline to Day 7 (1.11 ± 0.98 to 0.66 ± 0.90) (*P* < 0.0001 for both). There were significant reductions in itching, size of hives, and hive score at both Days 7 and 14. Treatment resulted in moderate, marked, or complete relief of symptoms in 76.2% of subjects. Desloratadine was well tolerated, with no adverse events reported.

**Conclusion:**

In an open-label, observational study, desloratadine 5 mg once daily significantly decreased symptoms of chronic idiopathic urticaria and improved subject QoL.

## Introduction

Chronic urticaria, or urticaria that lasts longer than 6 weeks, is a common and frustrating condition [1]. Chronic idiopathic urticaria, or chronic urticaria of undefined etiology, occurs in about 70% of patients diagnosed with chronic urticaria and affects about 0.1% to 3% of the general population [2–4]. Because classification of urticaria is complex and includes a spectrum of clinical manifestations and subtypes, recent guidelines do not distinguish between chronic urticaria and chronic idiopathic urticaria. Given that the differential diagnosis is difficult and treatment approaches do not differ greatly, chronic urticaria may become the preferred term in the future [5,6]. The idiopathic nature of chronic idiopathic urticaria has been curtailed by the growing appreciation of the role of autoantibodies against IgE and the FcεRI receptor in the pathophysiology of the disease [7–11].

The effect of chronic idiopathic urticaria on the patient depends on the duration and severity of the condition [1]. Up to 20% of patients with chronic idiopathic urticaria have symptoms for 10 to 20 years [12,13]. Patients can be greatly affected by the recurrent itch and physically unappealing appearance of the lesions associated with chronic idiopathic urticaria [1,14]. Other factors, such as anxiety induced by the continual relapses, uncertainty of the cause, and negative effects on social life, may also contribute to a decline in quality of life (QoL) [15]. The profound effect of the physical symptoms of chronic idiopathic urticaria on patients' emotions and daily functioning is partly due to sleep disruption [1,14,16–18]. This can lead to fatigue, loss of energy, and physical and mental disturbance [14]. Despite the fact that chronic idiopathic urticaria interferes with patients' sense of well-being and QoL [16,19], physicians generally underestimate the condition's effect on their patients' lives [15].

Controlled trials have demonstrated the efficacy of antihistamines in the treatment of chronic idiopathic urticaria [20]. Second-generation antihistamines are the recommended first-line therapy for chronic idiopathic urticaria [6]. Desloratadine, a newer, nonsedating, second-generation antihistamine, is effective, safe, and provides rapid onset of action and long duration of symptom relief while improving QoL [1,21,22].

This office-based trial was designed to study desloratadine therapy for chronic idiopathic urticaria in the real-world setting of everyday clinical practice (and the type of patient population normally seen in such a practice). It employed a chronic idiopathic urticaria-specific instrument, the Aerius^1^ Quality of Life Questionnaire (AEQLQ) (Table 1), and patient diaries to assess the effect of desloratadine 5 mg once daily on chronic idiopathic urticaria symptoms, disease severity, daily activities, and patient QoL.

**Table 1:** Aerius Quality of Life Questionnaire (AEQLQ)

The aim of this questionnaire is to measure how your skin problem has affected your life OVER THE PAST WEEK. Please tick ✓ one box for each problem.	
OVER THE PAST WEEK …	
Symptoms	
1.Have you been bothered by symptoms, such as itchiness, pain, soreness, or a stinging sensation?	
Very much	__3
A lot	__2
A little	__1
Not at all	__0
2.Have your skin symptoms interfered with the quality or length of your sleep?	
Very much	__3
A lot	__2
A little	__1
Not at all	__0
Interference with usual activities	
3.Have your skin symptoms interfered with your outdoor activities?	
Very much	__3
A lot	__2
A little	__1
Not at all	__0
4.Have your skin problems affected your ability to participate in sports or other physical activities?	
Very much	__3
A lot	__2
A little	__1
Not at all	__0
5.Have your skin problems affected your ability to participate in leisure or other social activities?	
Very much	__3
A lot	__2
A little	__1
Not at all	__0
6.Have your skin problems interfered with your ability to work or study?	
Very much	__3
A lot	__2
A little	__1
Not at all	__0
Social aspects	
7.Are you embarrassed or self-conscious about your skin?	
Very much	__3
A lot	__2
A little	__1
Not at all	__0
8.Have your skin problems created problems with your partner, close friends, or relatives?	
Very much	__3
A lot	__2
A little	__1
Not at all	__0
9.Have your skin problems interfered with your ability to engage in or enjoy sexual activities?	
Very much	__3
A lot	__2
A little	__1
Not at all	__0
10.Have your skin problems influenced how you dress?	
Very much	__3
A lot	__2
A little	__1
Not at all	__0
Please check you have answered EVERY question. Thank you.	

## Methods

### Study Design

This open-label, 2-week study evaluated the impact of desloratadine on QoL, symptom relief, and improvement in disease severity in a nonrandomized setting. Physicians from 157 medical centers across Canada could recruit up to 5 subjects each. Subjects were required to complete 2 visits: one at baseline (Day 1) and another at Day 14. Subjects evaluated their QoL at baseline, Day 7, and Day 14 using the AEQLQ. Diaries were provided, and subjects were instructed to make daily entries on time of medication dosing, interference with sleep (morning only), and interference with daily outdoor activities (evening only), and to assess twice daily severity of pruritus, number of hives, and size of the largest hive.

Subjects who completed the questionnaires and the diary received a $25 gift certificate for their participation. Physicians received $100 per subject enrolled. Medication was supplied free of charge.

This study was conducted under the auspices of a centralized institutional review board, in accordance with the principles of the 1974 Declaration of Helsinki and subsequent amendments. All subjects provided written informed consent.

### Subjects and Treatment

Subjects were eligible to enroll if they were ≥18 years of age; were diagnosed with chronic idiopathic urticaria; and had a hive score of ≥1, a pruritus score of ≥2, and an overall condition score of ≥2 (see below for scoring system). Inclusion criteria included a 6-week history of chronic idiopathic urticaria and a current flare of at least 3-weeks' duration (hives present at least 3 days per week). Subjects were excluded if they were pregnant or breastfeeding; had documented drug or food allergies; had other known etiologic factors contributing to their urticaria; or had a history of hypersensitivity to desloratadine or any of its excipients. Subjects were instructed to take one 5-mg desloratadine tablet daily at approximately the same time in the morning for 14 days. Concomitant medications for chronic idiopathic urticaria, including topical creams and other antihistamines, were reviewed and recorded by the physician at baseline and final visit. The most common concomitant medications recorded at baseline were cetirizine (taken by 24.1% of subjects), diphenhydramine (11.5%), and hydroxyzine HCl (11.2%); the most common concomitant medications recorded at final visit (other than desloratadine) were cetirizine (15.1% of subjects), hydroxyzine HCl (14.3%), and diphenhydramine (7.1%). Subjects could adjust concomitant medication dosing as required.

### Efficacy and Safety Assessments

Symptom scores were rated jointly by physicians and subjects at baseline and through review of subjects' daily diaries at final visit, reflecting real-world clinical practice in which patients and physicians estimate disease severity in concert. Evaluations included severity of itching, hive score, and overall condition. Quality of life was assessed using the AEQLQ, which consists of 10 equally weighted questions (severity of symptoms, interference with sleep, interference with outdoor activities, ability to participate in sports/physical activities, ability to participate in leisure/social activities, ability to work or study, feelings of self-consciousness, problems with partner/close friends/relatives, sexual dysfunction, and influence on dress). This questionnaire is based on the Dermatology Life Quality Index (DLQI), a validated measure of QoL in subjects with skin diseases [23]; the AEQLQ substitutes a question on sleep for the DLQI's question on disease treatment. Subjects completed the AEQLQ at baseline, Day 7, and Day 14.

Symptom relief and improvement in disease severity were evaluated using subjects' daily diaries. Subjects recorded the time medication was taken daily; a self-assessment evaluation (pruritus, number of hives, size of largest hive) twice daily; interference with sleep once daily in the morning; and interference with daily activities once daily in the evening. Pruritus was rated on a scale of 0 to 3, with 0 being not present and 3 being the most severe. Hives were rated on a scale of 0 to 4, with 0 representing no hives and 4 representing ≥20 hives. The size of the largest hive was rated on a scale of 0 to 3, with 0 representing no hives and 3 representing hives >2.5 cm. Interference with sleep or daily activities was rated on a scale of 0 to 3, with 0 representing none and 3 representing severe disturbances.

At the final visit, physicians and subjects jointly assessed response to treatment according to the following classifications: complete relief, marked relief, moderate relief, slight relief, or treatment failure. Physicians were instructed to report all adverse events (AEs). Adverse events were monitored by the treating physician and followed to satisfactory resolution or stabilization. Adverse events were defined as any unfavorable and unintended sign, symptom, or disease associated with the use of a medicinal product, whether it was considered related to the medicinal product; occurring in the course of the use of a drug, biological product, or device; associated with, or observed in conjunction with, product overdose, whether accidental or intentional; associated with, or observed in conjunction with, product abuse; and/or associated with, or observed in conjunction with, product withdrawal. Serious AEs were considered to be any AE occurring at any dose that resulted in death, a life-threatening adverse drug experience, inpatient hospitalization, a persistent or significant disability/incapacity, or a congenital anomaly or birth defect.

### Statistical Analysis

In this single-cohort, repeated-measure, observational study, self-reported nominal/ordinal dependent variables were treated as continuous variables; the different severity categories (from none to severe) were assigned and parametric statistical methods were performed on the main score. A repeated-measure analysis of variance (ANOVA) was used for the primary outcome variables (symptom evaluation, global response, and QoL). These test whether or not the observed change from baseline to Day 14 is likely to be due to chance (i.e., a random effect). Post hoc, pair-wise comparisons were also performed for analysis time points. To evaluate the impact of concomitant medication on outcome parameters, a mixed-model ANOVA analysis was used. No adjustment for multiple comparisons was performed.

## Results

**Figure 1:**
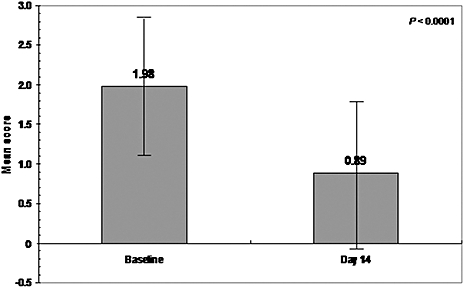
Combined physician and subject itching score (0–3 scale) at baseline and final visit.

Subject (N = 348) baseline characteristics are outlined in Table 2. In 327 subjects who completed the study, desloratadine significantly decreased itching scores from baseline to Day 14 (1.98 ± 0.87 vs. 0.89 ± 0.90; *P* < 0.0001), as evaluated by physicians and subjects by discussion at baseline and by reviewing diary entries at Day 14 (Figure 1; Table 3). According to subjects' daily diary entries, desloratadine treatment resulted in significant reductions in itching at Days 7 and 14 from baseline (1.34 ± 1.01 at baseline vs. 0.73 ± 0.92 at Day 7 and 0.90 ± 0.96 at Day 14; *P* < 0.0001) (Table 4). There was an additional significant reduction in itching from Day 7 to Day 14 (*P* = 0.0101).

**Table 4:** Subject diary ratings

Symptom (range of score)	Baseline	Day 7	Day 14
Itching (0–3)*			
Day	1.34 ± 1.01	0.73 ± 0.92	0.90 ± 0.96
Night	1.58 ± 0.98	0.84 ± 0.99	0.96 ± 1.01
Size of hives (0–3)†			
Day	1.28 ± 1.09	0.83 ± 0.94	0.97 ± 1.03
Night	1.53 ± 1.07	1.07 ± 1.09	0.93 ± 1.02
Number of hives (0–4)‡			
Day	1.81 ± 1.49	1.13 ± 1.40	1.38 ± 1.47
Night	2.13 ± 1.51	1.21 ± 1.43	1.42 ± 1.51
Interference with sleep (0–3)§			
Morning	0.99 ± 1.10	0.57 ± 0.90	0.49 ± 0.84
Interference with daily activities (0–3)¶			
Evening	0.93 ± 0.88	0.49 ± 0.82	0.62 ± 0.90

* *P* < 0.0001 baseline vs. Day 7 and Day 14; *P =*0.0101 Day 7 vs. Day 14. (am); *P =*0.0701 Day 7 vs. Day 14 (pm).

† *P* < 0.0001 baseline vs. Day 7 and Day 14; *P* = 0.0329 Day 7 vs. Day 14; *P =*0.0181 Day 7 vs. Day 14 (pm).

‡ *P* < 0.0001 baseline vs. Day 7 and Day 14; *P* = 0.0043 Day 7 vs. Day 14; *P =*0.0102 Day 7 vs. Day 14 (pm).

§ *P* < 0.0001 baseline vs. Day 7 and Day 14; *P* = 0.2302 Day 7 vs. Day 14.

¶ *P* < 0.0001 baseline vs. Day 7 and Day 14; *P =*0.0096 Day 7 vs. Day 14.

**Table 3:** Main efficacy results

Evaluation (range of score)	Baseline	Day 7	Day 14	*P* value
Symptom				
Itching (0–3)	1.98 ± 0.87	—	0.89 ± 0.90	<0.0001
Hive score (0–4)	2.12 ± 1.44	—	1.02 ± 1.31	<0.0001
Overall condition (0–3)	2.19 ± 0.66	—	1.14 ± 0.89	<0.0001
Quality of life (0–3)				
Bothered by CIU symptoms	2.08 ± 0.83	1.36 ± 0.87	1.17 ± 0.91	<0.0001
CIU interfered with sleep	1.42 ± 1.03	0.85 ± 0.89	0.68 ± 0.83	<0.0001
CIU interfered with outdoor activity	1.11 ± 0.98	0.66 ± 0.90	0.56 ± 0.87	<0.0001
CIU interfered with sports	0.90 ± 0.97	0.59 ± 0.88	0.48 ± 0.86	<0.0001
CIU interfered with leisure/social	1.05 ± 1.01	0.62 ± 0.88	0.49 ± 0.82	<0.0001
CIU interfered with work/study	1.11 ± 0.96	0.65 ± 0.88	0.56 ± 0.82	<0.0001
Embarrassed, self–conscious about skin	1.55 ± 1.05	1.03 ± 1.01	0.88 ± 0.97	<0.0001
CIU created problems with partner	0.55 ± 0.82	0.35 ± 0.67	0.30 ± 0.63	<0.0001
CIU interfered with sexual activities	0.64 ± 0.95	0.47 ± 0.87	0.35 ± 0.75	<0.0001
CIU influenced dressing	1.33 ± 1.09	0.96 ± 1.04	0.80 ± 0.99	<0.0001
Global response to therapy (0–5)	—	—	2.59 ± 1.33	—

**Table 2:** Study population: subject characteristics (N = 348)

		Mean ± SD
Age (years)		44.2 ± 14.9
Number of months subject had suffered from chronic idiopathic urticaria		35.2 ± 71.5
Duration of current flare (months)		4.4 ± 5.6

	n	%

Female	244	70.1%
Subjects taking **NO** concomitant medications at baseline	70	20.1%
Subjects taking **NO** concomitant antihistamine at baseline	122	35.1

Desloratadine treatment also resulted in significant reductions in the size of hives and hive scores at Days 7 and 14 from baseline (*P* < 0.0001). Improvements were observed early during treatment and continued to Day 14.

Subject diary entries showed that interference with sleep had significant improvements by Day 7 and Day 14 (*P* < 0.0001). Interference with daily outdoor activities also significantly improved by Day 7 and Day 14 (*P* < 0.0001).

Desloratadine significantly decreased subjects' overall condition (combined score for itching and number of hives) from baseline to Day 14 (2.19 ± 0.66 vs. 1.14 ± 0.89; *P* < 0.0001). Subjects reported itching and number of hives as twice-daily diary entries. Physicians and subjects jointly determined itching score by discussion at baseline and by review of subject diary entries at Day 14. Desloratadine treatment also significantly decreased scores for all 10 AEQLQ measures from baseline to Day 7 and to Day 14 (*P* < 0.0001). Incremental significant decreases were observed from Day 7 to Day 14 for all domains on the AEQLQ except “created problems with partner.” Sleep interference scores were reduced by 40% from baseline to Day 7 (1.42 ± 1.03 vs. 0.85 ± 0.89) and 52% from baseline to Day 14 (1.42 ± 1.03 vs. 0.68 ± 0.83). Interference with work/study scores showed a 41% decrease from baseline to Day 7 and a 50% decrease from baseline to Day 14. Desloratadine treatment resulted in moderate (21.7%), marked (30.3%), or complete (24.2%) relief of symptoms.

In this study, desloratadine was well tolerated, with no AEs reported. A total of 21 subjects were lost to follow-up.

## Discussion

Treatment for urticaria begins with the elimination of any known causative or exacerbating factors (i.e., cold, light, or pressure); therefore, taking a comprehensive patient history is paramount. However, as the causes of nonphysical or autoimmune-based chronic urticaria are by definition unknown, treatment typically focuses on symptom relief.

The most common approach to managing chronic urticaria is to prevent the release of histamine or to block its effects at receptor sites on nerves and endothelial cells. Therefore, H_1_ antihistamines are the cornerstone of urticaria treatment. First-generation antihistamines (e.g., brompheniramine, diphenhydramine, and ketotifen) are effective, but they are also associated with AEs caused by their lack of selectivity for the H_1_ receptor (e.g., antimuscarinic effects, appetite stimulation, weight gain, gastrointestinal effects), as well as their binding to cerebral H_1_receptors, which causes central nervous system effects, such as somnolence and cognitive impairment. Newer, second-generation agents, such as desloratadine, are therefore generally preferred as first-line therapy for chronic urticaria due to their proven efficacy and favorable safety profiles [24].

Although chronic urticaria occasionally resolves spontaneously, symptoms can potentially last for many years. The prolonged duration of the disease can have a profoundly negative impact on a patient's sense of well-being. Long-lasting, safe, and effective second-generation antihistamines have been shown to substantially improve patient QoL and, therefore, remain the first choice for chronic urticaria therapy.

In the current study, treatment with desloratadine resulted in rapid improvements across all measured QoL domains. Significant improvements were observed by Day 7 in all 10 measured AEQLQ domains, and continued improvement was seen on Day 14. Desloratadine reduced itching during the night, interference with sleep, and interference with daily activities from Day 1 through the end of the 14-day study period as assessed by subjects in their daily diaries.

## Limitations

The QoL tool used in this study, the AEQLQ, is based on and derived from the validated QoL instrument DLQI, which has been widely used in dermatology across a variety of disease states. Studies of desloratadine that are similar to the one outlined in this paper have used the DLQI and have been published in peer-reviewed literature. We have verified that the AEQLQ, although derived from a validated tool, has neither been validated nor been cited previously. There were several limitations in the study design. The study used a single agent in an open-label design and was not placebo controlled; however, an open-label design in a patient population normally seen in clinical practice more closely approximates a physician's everyday experience. In addition, multiple statistical comparisons were made without adjustment, and no data were available for the 21 subjects who were lost to follow up.

## Conclusion

In this open-label, multicenter, uncontrolled observational study in 348 subjects with chronic idiopathic urticaria, desloratadine 5 mg once daily significantly improved QoL, pruritus, hive size and number, and overall condition. Improvements were noted at Day 7 and Day 14.

## Footnotes

1 Desloratadine is marketed as Aerius® (Schering-Plough, Kenilworth, NJ, USA) in Europe and Canada.

## References

[1] Lachapelle JM, Decroix J, Henrijean A, Roquet-Gravy PP, De Swerdt A, Boonen H (2006). Desloratadine 5 mg once daily improves the quality of life of patients with chronic idiopathic urticaria. J Eur Acad Dermatol Venereol.

[2] Champion RH (1988). Urticaria: Then and now. Br J Dermatol.

[3] Strachan DD Urticaria, chronic. http://www.emedicine.com/derm/topic443.htm.

[4] Sabroe RA, Greaves MW (1997). The pathogenesis of chronic urticaria. Arch Dermatol.

[5] Zuberbier T, Bindslev-Jensen C, Canonica W, Grattan CEH, Greaves MW, Henz BM (2006). EAACI/GA^2^LEN/EDF guideline: Definition, classification and diagnosis of urticaria. Allergy.

[6] Zuberbier T, Bindslev-Jensen C, Canonica W, Grattan CEH, Greaves MW, Henz BM (2006). EAACI/GA^2^LEN/EDF guideline: Management of urticaria. Allergy.

[7] Gruber BL, Baeza ML, Marchese MJ, Agnello V, Kaplan AP (1988). Prevalence and functional role of anti-IgE autoantibodies in urticarial syndromes. J Invest Dermatol.

[8] Grattan CEH, Francis DM, Hide M, Greaves MW (1991). Detection of circulating histamine releasing autoantibodies with functional properties of anti-IgE in chronic urticaria. Clin Exp Allergy.

[9] Hide M, Francis DM, Grattan C, Hakimi J, Kochan JP, Greaves MW (1993). Autoantibodies against the high-affinity IgE receptor as a cause of histamine release in chronic urticaria. N Engl J Med.

[10] Zweiman B, Valenzano M, Atkins PC, Tanus T, Getsy JA (1996). Characteristics of histamine-releasing activity in the sera of patients with chronic idiopathic urticaria. J Allergy Clin Immunol.

[11] Tong LJ, Balakrishnan G, Kochan JP, Kinét J-P, Kaplan AP (1997). Assessment of autoimmunity in patients with chronic urticaria. J Allergy Clin Immunol.

[12] Sheikh J Urticaria. http://www.emedicine.com/med/topic3014.htm.

[13] Greaves M (2000). Chronic urticaria. J Allergy Clin Immunol.

[14] O'Donnell BF, Lawlor F, Simpson J, Morgan M, Greaves MW (1997). The impact of chronic urticaria on the quality of life. Br J Dermatol.

[15] Grob J-J, Gaudy-Marqueste C (2006). Urticaria and quality of life. Clin Rev Allergy Immunol.

[16] Baiardini I, Giardini A, Pasquali M, Dignetti P, Guerra L, Specchia C (2003). Quality of life and patients' satisfaction in chronic urticaria and respiratory allergy. Allergy.

[17] Staubach P, Eckhardt-Henn A, Dechene M, Vonend A, Metz M, Magerl M (2006). Quality of life in patients with chronic urticaria is differentially impaired and determined by psychiatric comorbidity. Br J Dermatol.

[18] Sabroe RA, Seed PT, Stat C, Francis DM, Barr RM, Kobza Black A (1999). Chronic idiopathic urticaria: Comparison of the clinical features of patients with and without anti-FcεRI or anti-IgE autoantibodies. J Am Acad Dermatol.

[19] Poon E, Seed PT, Greaves MW, Kobza-Black A (1999). The extent and nature of disability in different urticarial conditions. Br J Dermatol.

[20] Lee EE, Maibach HI (2001). Treatment of urticaria: An evidence-based evaluation of antihistamines. Am J Clin Dermatol.

[21] Ring J, Hein R, Gauger A, Bronsky E, Miller B, and the Desloratadine Study Group (2001). Once-daily desloratadine improves the signs and symptoms of chronic idiopathic urticaria: A randomized, double-blind, placebo-controlled study. Int J Dermatol.

[22] Monroe E, Finn A, Patel P, Guerrero R, Ratner P, Bernstein D, Desloratadine Urticaria Study Group (2003). Efficacy and safety of desloratadine 5 mg once daily in the treatment of chronic idiopathic urticaria: A double-blind randomized, placebo-controlled trial. J Am Acad Dermatol.

[23] Finlay AY, Khan GK (1994). Dermatology Life Quality Index (DLQI)—A simple practical measure for routine clinical use. Clin Exp Dermatol.

[24] Thompson AK, Finn AF, Schoenwetter WF (2000). Effect of 60 mg twice-daily fexofenadine HCl on quality of life, work and classroom productivity, and regular activity in patients with chronic idiopathic urticaria. J Am Acad Dermatol.

